# The potential role of appetite in predicting weight changes during treatment with olanzapine

**DOI:** 10.1186/1471-244X-10-72

**Published:** 2010-09-14

**Authors:** Michael Case, Tamas Treuer, Jamie Karagianis, Vicki Poole Hoffmann

**Affiliations:** 1 Lilly USA, LLC, Indianapolis, IN, USA; 2 Eli Lilly & Company, Budapest, Hungary, EU; 3 Eli Lilly Canada Inc., Toronto, Ontario, Canada and Memorial University of Newfoundland, St. John's, Newfoundland and Labrador, Canada

## Abstract

**Background:**

Clinically significant weight gain has been reported during treatment with atypical antipsychotics. It has been suggested that weight changes in patients treated with olanzapine may be associated with increased appetite.

**Methods:**

Data were used from adult patients for whom both appetite and weight data were available from 4 prospective, 12- to 24-week clinical trials. Patients' appetites were assessed with Eating Behavior Assessment (EBA, Study 1), Platypus Appetite Rating Scale (PARS, Study 2), Eating Inventory (EI, Study 3), Food Craving Inventory (FCI, Study 3), and Eating Attitude Scale (EAS, Study 4).

**Results:**

In Studies 1 (EBA) and 4 (EAS), patients who reported overall score increases on appetite scales, indicating an increase in appetite, experienced the greatest overall weight gains. However, in Studies 2 (PARS) and 3 (EI, FCI), patients who reported overall score increases on appetite scales did not experience greater weight changes than patients not reporting score increases. Early weight changes (2-4 weeks) were more positively correlated with overall weight changes than early or overall score changes on any utilized appetite assessment scale. No additional information was gained by adding early appetite change to early weight change in correlation to overall weight change.

**Conclusions:**

Early weight changes may be a more useful predictor for long-term weight changes than early score changes on appetite assessment scales.

**Clinical Trials Registration:**

This report represents secondary analyses of 4 clinical studies. Studies 1, 2, and 3 were registered at http://clinicaltrials.gov/ct2/home, under NCT00190749, NCT00303602, and NCT00401973, respectively. Study 4 predates the registration requirements for observational studies that are not classified as category 1 observational studies.

## Background

Treatment with atypical antipsychotics has been temporally associated with weight gain. Hypotheses about the potential mechanism have included direct effects of the known receptor affinities of each compound [1,2], effects on gastric and intestinal hormones [3], direct or indirect effects on the feeding and satiety centers in the brain [4], disturbance of the hypothalamus-pituitary-adrenal (HPA) axis [5], direct effect on insulin sensitivity [6], decrease in physical activity, and decrease in metabolic rate [7].

The extent of weight change and changes in metabolic parameters during treatment with antipsychotics varies between drugs. These variations may be due to differences in receptor pharmacology [8]. Kroeze et al. demonstrated that affinity to the histamine H1 receptor predicts weight gain associated with typical and atypical antipsychotics [9]. Olanzapine and clozapine both have high affinities for the 5-HT2C and the histamine H1 receptors, while antagonism of peripheral M3 muscarinic receptor and effects on central 5-HT2C may potentially be related to treatment-emergent diabetes observed independent of obesity.

While potential mechanisms for weight gain have been widely studied, the role of changes in appetite remains poorly understood. It is well known that executive functions are necessary to successfully manage eating behavior, and their impairment and disturbed weight regulation are often observed in patients with schizophrenia treated with antipsychotics. A recent pilot study showed that a delay of gratification and executive performance in individuals with schizophrenia may play a putative role for eating behavior and body weight regulation [10]. Additionally, increasing evidence suggests that general obesity is linked to adverse neurocognitive outcomes. Altered cognitive functions can independently affect the control of appetite [11]. Treatment with both clozapine and olanzapine have been temporally associated with food craving and binge eating [12,13].

Previous studies have observed that patients treated with atypical antipsychotics are more reactive to external eating cues as measured by the Three Factors Eating Behavior Questionnaire and the Dutch Eating Behavior Questionnaire [14]. Based on the observation of an association between weight gain and lack of cognitive restraint in the presence of increased appetite, it has been suggested that psychoeducational counseling in conjunction with adjunctive pharmacotherapeutic agents might limit weight gain during antipsychotic drug therapy [15].

An understanding of the role of appetite changes in weight gain during antipsychotic treatment would be helpful to clinicians and patients, some of whom report substantially increased appetite starting after their first dose of an antipsychotic.

Changes in appetite might serve as early warning signs of risk of weight gain as well as inform treatment decisions. If specific changes in appetite can be expected, patients can be informed in advance and may be better able to manage them. Here we test the hypothesis that changes in appetite might be indicative of a patient's weight gain during treatment with olanzapine.

## Methods

Presented are secondary analyses examining potential associations between changes in appetite and weight changes during treatment with olanzapine. The primary study objectives have been reported elsewhere [16-19]. The study protocols were reviewed and approved by individual institutional review boards prior to enrolling any patients, and the analyses presented here are consistent with the original ethics approvals. The studies were consistent with Good Clinical Practices and all applicable regulatory requirements. All participants provided written informed consent before receiving study therapy or undergoing study procedures.

### Study design

Included in the analyses were patients from 4 prospective, phase IV clinical trials examining the efficacy and safety of olanzapine in adult (18 to 65 years old in Studies 1, 2, and 3, ≥ 18 years old in Study 4) male and female patients diagnosed with schizophrenia, schizoaffective disorder, related psychosis, or bipolar disorder. In Study 1, patients received double-blind oral olanzapine 5-20 mg once daily (QD) for 12 weeks [16]. In Study 2, patients received double-blind oral olanzapine 5-20 mg QD for 16 weeks [18]. In Study 3, patients received open-label oral olanzapine 5-20 mg QD for 22 weeks [19]. Study 4 was an observational study in which patients received oral olanzapine at doses determined by the investigator as appropriate for the individual patient for 6 months (Table 1) [17]. Detailed inclusion and exclusion criteria can be found in the primary study reports [16-19].

**Table 1:** Summary of Study Designs

	Study 1	Study 2	Study 3	Study 4
**Patient age (years)**	18 to 65	18 to 65	18 to 65	≥ 18

**Study design**	Double-blind	Double-blind	Open-label	Observational

**Olanzapine dose (mg)**	5 to 20 mg QD	5 to 20 mg QD	5 to 20 mg QD	Determined by the investigator

**Adjunctive pharmacotherpay**	no	no	no or Amantadine 100 mg BID or Metformin 500 mg BID	no

**Appetite assessment scale**	Eating Behavior Assessment	Platypus Appetite Rating Scale	Eating Inventory and Food Craving Inventory	Eating Attitude Scale

**Dietary counseling**	yes	no	yes	no

**Study length (weeks)**	12	16	22	24

Abbreviations: BID = twice daily; QD = once daily.

### Clinical assessment of appetite

Across all 4 studies, appetite was assessed with 5 different scales: Eating Behavior Assessment (EBA, a Lilly-developed scale, assessing appetite and eating behavior with 9 standardized questions, grading responses on a scale from 0 to 4, where 0 = not at all and 4 = extremely; not validated; Study 1); Platypus Appetite Rating Scale (PARS, a Lilly-developed visual analog scale; not validated; Study 2); Eating Inventory (EI, Study 3) [20]; Food Craving Inventory (FCI, Study 3) [21]; and Eating Attitude Scale (EAS, a Lilly-developed scale, assessing appetite and eating behavior during the past 4 weeks with 10 standardized categories; not validated; Study 4) (Table 1).

### Statistical analysis

For each study, only patients for whom weight and appetite data at baseline, at 2 weeks (Study 4, 4 weeks), and at ≥ 1 later visit were available, were included in our analyses. Patients were assigned to distinct groups based on their overall and 2-week (Study 4, 4-week) appetite scale item scores and total scores. Score increase was defined as: positive value on EBA, > +5 units on PARS, > +1 unit on EI, > +1 unit on FCI, or > 0 units on EAS. No change in score was defined as: 0 units on EBA, ≥ -5 to ≤ +5 units on PARS, ≥ -1 to ≤ +1 units on EI, ≥ -1 to ≤ +1 units on FCI, or 0 units on EAS. Score decrease was defined as: negative value on EBA, < -5 units on PARS, < -1 unit on EI, < -1 unit on FCI, or < 0 units on EAS. For each group, mean overall weight change and mean appetite scale score changes were determined using observed case analyses. Additionally, to test the hypothesis of a linear trend between appetite and weight changes (i.e. greater increases in appetite are associated with greater increases in weight), pair-wise comparisons of mean weight changes in the "decrease" versus "no change" and the "no change" versus "increase" appetite groups were conducted. If both of these tests were significant and the magnitudes of the changes followed the hypothesized pattern, a linear trend would be suggested.

Additionally, several Pearson correlation coefficients were assessed and tested for statistical significance: a) between weight changes from baseline to endpoint and score changes on appetite scales from baseline to 2 weeks (Study 4, 4 weeks); b) between weight changes from baseline to endpoint and changes on appetite scales from baseline to endpoint; c) between baseline to endpoint weight changes and 2-week (Study 4, 4-week) weight changes; and d) between overall weight change and 2-week appetite scale changes, adjusted by 2-week weight change (the correlation of appetite changes on the residuals from the regression of endpoint weight changes on 2-week weight changes).

## Results

### Patients

Baseline demographic data for all patients included in our analyses are presented in Table 2. The distribution of patient ethnicities was different across all 4 studies. Study 1 included a majority of African American patients, while Studies 2 and 3 included mainly white patients, and the majority of patients in Study 4 self-identified as East and Southeast Asians.

**Table 2:** Baseline Demographics and Clinical Characteristics

Parameter	Study 1 (N = 68)	Study 2 (N = 65)	Study 3 (N = 50)	Study 4 (N = 622)
Age (years), mean (SD)	43.5 (9.5)	38.7 (12.2)	38.5 (12.0)	35.6 (12.2)

Male gender, n (%)	45 (66.2)	33 (50.8)	120 (60.3)	269 (43.2)

Ethnicity, n (%)				

White	27 (39.7)	36 (55.4)	87 (43.7)	148 (23.8)

African American	34 (50.0)	4 (6.2)	16 (8.0)	0

East/Southeast Asian	1 (1.5)	1 (1.5)	39 (19.6)	369 (59.3)

Native American	0	0	0	0

Hispanic	4 (5.9)	23 (35.4)	52 (26.1)	83 (13.3)

West Asian	0	0	4 (2.0)	1 (0.2)

Other	2 (2.9)	1 (1.5)	0	0

Native American/First Nation	0	0	1 (0.5)	0

Missing	0	0	0	21 (3.4)

Weight (kg), mean (SD)	86.3 (16.8)	81.2 (17.0)	77.5 (16.6)	64.1 (12.5)

BMI, mean (SD)	28.7 (5.1)	28.3 (4.8)	27.1 (4.7)	23.2 (3.9)

Appetite, mean (SD)	EBA Item #1: 1.5 (1.1)^a^	PARS: 65.7 (19.2)	EI-Cognitive Restraint: 7.6 (5.2)^b^	EAS 1: 1.6 (1.2)^c^
	EBA Item #2: 1.6 (1.1)^a^		EI-Disinhibition: 8.7 (4.6)^b^	EAS 2: 1.6 (1.1)^c^
	EBA Item #3: 1.1 (1.2)^a^		EI-Hunger: 7.9 (4.5)^b^	EAS 5: 2.3 (1.2)^d^
	EBA Item #4: 0.9 (1.1)^a^		FCI Total: 65.4 (20.1)	EAS 6: 1.3 (1.2)^f^
	EBA Item #5: 2.5 (1.0)^a^			EAS 7: 1.2 (1.2)^f^
	EBA Item #6: 0.9 (1.2)^a^			EAS 8: 0.7 (0.9)^f^
	EBA Item #7: 0.9 (1.2)^a^			EAS 9: 0.6 (0.9)^f^
	EBA Item #8: 0.4 (0.9)^a^			
	EBA Item #9: 0.1 (0.3)^a^			

Abbreviations: BMI = Body Mass Index; EAS = Eating Attitude Scale; EAS 1 = More hungry than usual; EAS 2 = Stronger appetite than usual; EAS 5 = Felt comfortably full when meal was finished; EAS 6 = It took an excessive amount of food to feel full; EAS 7 = Thoughts were preoccupied with food; EAS 8 = Ate until uncomfortably full; EAS 9 = Could not stop eating; EB = Eating Behavior Assessment; EI = Eating Inventory; FCI = Food Craving Inventory; kg = kilograms; N = number of patients in study included in the current analyses; n = number of patients affected; PARS = Platypus Appetite Rating Scale; SD = standard deviation;

^a ^n = 68; ^b ^only assessed in patients in the United States, n = 17; ^c ^n = 606; ^d ^n = 602; ^e ^n = 604; ^f ^n = 605

### Weight changes

In all 4 studies, patients experienced statistically significant (p < .05) mean weight increases from baseline to endpoint (Study 1: 86.3 kg at baseline, 89.6 kg at endpoint; Study 2: 81.2 kg at baseline, 84.1 kg at endpoint; Study 3: 85.4 kg at baseline, 90.8 kg at endpoint; Study 4: 64.1 kg at baseline, 68.3 kg at endpoint).

### Appetite changes

An increase in patients' appetite from baseline to endpoint was observed in Study 1 (EBA item #1: 1.5 at baseline, 1.7 at endpoint, p = .21; EBA item #2: 1.6 at baseline, 1.6 at endpoint, p = .72; EBA item #3: 1.1 at baseline, 1.2 at endpoint, p = .11; EBA item #4: 0.9 at baseline, 1.1 at endpoint, p = .22; EBA item #5: 2.5 at baseline, 2.5 at endpoint, p = .35; EBA item #6: 0.9 at baseline, 1.1 at endpoint, p = .42; EBA item #7: 0.9 at baseline, 1.0 at endpoint, p = .32; EBA item #8: 0.4 at baseline, 0.6 at endpoint, p = .36; EBA item #9: 0.1 at baseline, 0.3 at endpoint, p = .12), while in Studies 2, 3, and 4, patients' appetites decreased in the course of the trials (Study 2 - PARS: 65.7 at baseline, 58.9 at endpoint, p = .04; Study 3 - EI cognitive restraint: 7.6 at baseline, 11.3 at endpoint, p = .09, EI disinhibition: 8.7 at baseline, 5.4 at endpoint, p = .16, EI hunger: 7.9 at baseline, 4.8 at endpoint, p = .17, FCI total: 65.4 at baseline, 61.5 at endpoint, p < .0001; Study 4 - EAS 1: 1.6 at baseline, 1.4 at endpoint, p < .0001, EAS 2: 1.6 at baseline, 1.4 at endpoint, p < .0001, EAS 5: 2.3 at baseline, 2.1 at endpoint, p < .0001, EAS 6: 1.3 at baseline, 1.1 at endpoint, p < .0001, EAS 7: 1.2 at baseline, 1.1 at endpoint, p < .0001, EAS 8: 0.7 at baseline, 0.5 at endpoint, p = .85, EAS 9: 0.6 at baseline, 0.5 at endpoint, p = .48).

### Associations between appetite scale score changes and weight changes

**Figure 1:**
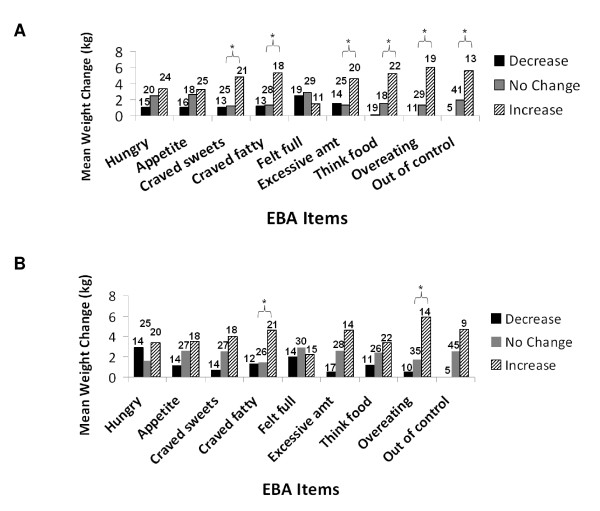
**Study 1 - A) Relationship of overall EBA item score changes and overall weight changes**. B) Relationship of 2-week EBA item score changes and overall weight changes. Abbreviations: EBA = Eating Behavior Assessment; kg = kilogram.

In Studies 1 (EBA) and 4 (EAS), score increases on single appetite assessment scale items, both at 2 or 4 weeks and at last measurement, indicating an increase in appetite, occurred in patients who experienced the greatest overall weight gains (Figures 1a+b, 2a+b). However, in Studies 2 (PARS) and 3 (EI, FCI), patients who reported score increases on appetite scales items at 2 weeks and/or at last measurement did not consistently experience greater weight changes than patients reporting no score changes or score decreases. The only individual appetite scale item that was correlated with later weight increase was an increase in the appetite for fatty fast food at 2 weeks in patients in Study 3 who showed the greatest overall weight change. Analysis of overall total score changes on appetite scales for Studies 1, 2, and 3 (no total score available for Study 4) showed that patients who experienced a decrease in total scores in appetite assessment scales had the lowest weight gains (≤ 1.6 kg).

**Figure 2:**
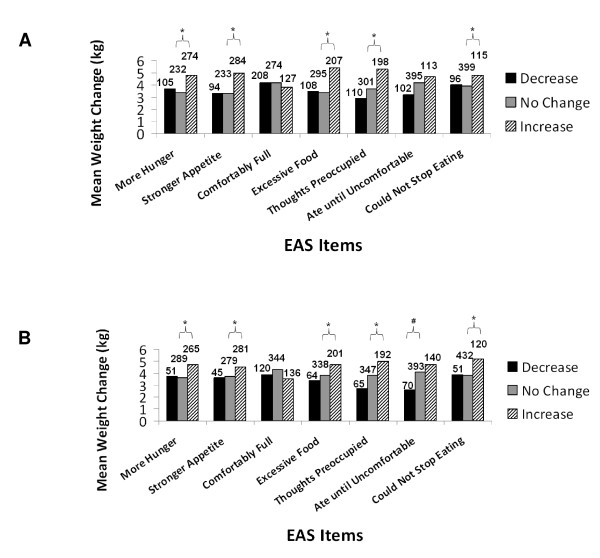
**Study 4 - A) Relationship of overall EAS item score changes and overall weight changes**. B) Relationship of 4-week EAS item score changes and overall weight changes. Abbreviations: EAS = Eating Assessment Scale; kg = kilogram

Statistically significant differences in the pair-wise comparisons among patient groups with distinct appetite rating scale scores were observed in Studies 1 (Figures 1a and 1b) and 4 (Figure 2a and 2b), where the "increase" appetite group showed significantly greater weight change than the "no change" appetite group on specific EBA and EAS items. However, the "decrease" appetite groups did not show significantly less weight change than the "no change" appetite groups on these same items.

### Correlation coefficients between appetite scale score changes and weight changes

In all 4 studies, early weight changes (2-4 weeks) had stronger correlations to overall weight changes than both overall and early (2-4 weeks) changes on any appetite scale examined (Table 3). Adjustment of early appetite scale changes by early weight changes demonstrated that early appetite scale assessments in conjunction with early weight changes do not provide additional information for predicting overall weight changes.

**Table 3:** Weight Changes and Appetite Scale Score Changes

	Overall Change^a^	2- or 4-Week Change^b^	2- or 4-Week Change - Adjusted^c^
Study 1 - Eating Behavior Assessment			

How hungry?	.304* (n = 59)	.058 (n = 59)	-.050 (n = 59)

Appetite?	.282* (n = 59)	.157 (n = 59)	.097 (n = 59)

Craved sweets?	.444 ***(n = 59)	.336** (n = 59)	.238 (n = 59)

Craved fatty?	.356** (n = 59)	.243 (n = 59)	.229 (n = 59)

Felt full?	-.062 (n = 59)	.048 (n = 59)	.039 (n = 59)

Ate excessive amount?	.277* (n = 59)	.240 (n = 59)	.117 (n = 59)

Thinking of food?	.427*** (n = 59)	.164 (n = 59)	.129 (n = 59)

Overeating?	.482*** (n = 59)	.345** (n = 59)	.209 (n = 59)

Out of control eating?	.493*** (n = 59)	.299* (n = 59)	.154 (n = 59)

Study 1 - Weight	1 (n = 59)	.533*** (n = 59)	N/A

Study 2 - Platypus Appetite Rating Scale	.141 (n = 65)	.090 (n = 63)	.101 (n = 63)

Study 2 - Weight	1 (n = 63)	.502*** (n = 63)	N/A

Study 3 - Eating Inventory^d^			

Cognitive Restraint	-.012 (n = 66)	.044 (n = 66)	.227 (n = 66)

Disinhibition	.138 (n = 66)	-.089 (n = 66)	-.084 (n = 66)

Hunger	-.046 (n = 66)	-.196 (n = 66)	-.139 (n = 66)

Study 3 - Food Craving Inventory			

Carbohydrates	-.063 (n = 188)	-.046 (n = 186)	-.061 (n = 186)

Fatty Fast Food	.019 (n = 188)	-.037 (n = 188)	-.032 (n = 188)

High Fat	.045 (n = 187)	.001 (n = 186)	-.029 (n = 186)

Sweets	-.017 (n = 188)	-.030 (n = 187)	-.048 (n = 187)

Total	.001 (n = 185)	-.024 (n = 184)	-.051 (n = 184)

Study 3 - Weight	1 (n = 189)	.507*** (n = 189)	N/A

Study 4 - Eating Attitude Scale			

More hungry than usual	.162*** (n = 611)	.153*** (n = 605)	.013 (n = 591)

Stronger appetite than usual	.198*** (n = 611)	.173*** (n = 605)	.023 (n = 591)

Felt comfortably full when meal was finished	-.018 (n = 609)	-.025 (n = 600)	-.041 (n = 591)

It took an excessive amount of food to feel full	.212*** (n = 610)	.119** (n = 603)	-.029 (n = 591)

Thoughts were preoccupied with food	.189*** (n = 609)	.199*** (n = 604)	.069 (n = 591)

Ate until uncomfortably full	.105** (n = 610)	.116** (n = 603)	-.001 (n = 591)

Could not stop eating	.019 (n = 610)	.106** (n = 603)	.027 (n = 591)

Study 4 - Weight	1 (n = 608)	.561*** (n = 608)	N/A

^a ^Pearson Correlation Coefficients between overall weight change and overall appetite scale changes or overall weight change

^b ^Pearson Correlation Coefficients between overall weight change and 2-week appetite scale changes or 2-week weight change; Study 4: Pearson Correlation Coefficients between overall weight change and 4-week appetite scale changes or 4-week weight change

**^c ^**partial Pearson Correlation Coefficients between overall weight change and 2-week appetite scale changes, adjusted by 2-week weight change (the correlation of appetite changes on the residuals from the regression of endpoint weight changes on 2-week weight changes)

*p < .05; **p < .01; ***p < .001

## Discussion

Our analyses demonstrate an inconsistent association between changes in appetite and weight change during treatment with olanzapine; results varied depending on study and appetite assessment scale used. Overall, early weight changes may be a more useful predictor of long-term weight changes compared with early score changes on appetite assessment scales. To our knowledge, this is the first study exploring a potential correlation between changes in appetite and weight changes during treatment with olanzapine.

Our observation that early weight changes correlate strongly with long-term weight changes is in agreement with earlier findings [22]. The absence of a consistent correlation between changes in appetite and weight changes was an unexpected finding, as one would expect that changes in appetite will result in changes in eating habits and consequently changes in weight. We cannot exclude the possibility that the appetite assessment scales might not have accurately measured appetite in our patient population. However, weight increase during treatment with olanzapine might not be associated with increased appetite. In experiments with female rats, hyperphagia and sedation were observed to occur concomitantly during exposure to olanzapine, two behaviors that interact competitively without necessarily increasing appetite [15,23]. However, earlier studies with sulpiride showed that there is no weight gain in female rats in the absence of hyperphagia [24]. Another reason for the inconsistency of our observations might be the possibility that weight gain during treatment with olanzapine may be associated with several biochemical mechanisms, which might manifest in a variety of clinical conditions accompanying weight gain [25].

The observed variations in associations between changes on appetite assessment scales and weight changes might also be due to inherent differences between the scales that were utilized and differences among the study populations. One such difference among study populations might be the extent of clinical improvement during therapy. While our analysis is limited by the lack of a subanalysis of clinical improvement versus appetite, it has been observed previously that clinical improvement of psychotic symptoms in patients with schizophrenia seems to coincide with increased food intake [26]. Interestingly, EBA and EAS, which showed within the examined assessment scales the greatest similarities with one another with regard to items included, were also most similar in their assessment results. EBA and EAS were the only appetite scales for which patients with a score increase indicating increased appetite consistently showed the greatest overall weight gains compared with patients with no score increase. Additionally, score increases of several EBA and EAS items that might indicate binge eating showed strong correlations with weight gain.

All correlation analyses were repeated using a 7% increase in weight (clinically significant weight gain) as cutoff point. The results from those analyses were in agreement with the presented data from analyses examining correlations between change in weight and change in appetite assessment scale scores.

Our analyses were limited by the differences in study design across the 4 studies that were utilized: differences included study length, numbers and geographic locations of participating sites (resulting in different patient ethnicities), previous antipsychotic exposure, timing of appetite assessment, co-treatment of some patients in Study 3 with amantadine or metformin, and blinding procedures. Additionally, in Studies 1 and 3 patients received dietary counseling to control potential weight gain, while patients did not receive dietary counseling in Studies 2 and 4. Interestingly, the strongest correlations between change in appetite and change in weight were observed in Study 1, which was also the shortest study included in the current analyses and the only study in which increased appetite was observed (12 weeks versus 16 to 24 weeks for Studies 2, 3, and 4). In Study 2, patients had to have already gained at least 5 kg or 1 unit of body mass index (BMI) before randomization; therefore, most appetite increase probably occurred before the study started, especially when considering that all patients had been receiving olanzapine for 6 to 54 weeks before the 2-week appetite assessment occurred. Consequently, comparisons between Study 2 and Studies 1, 3, and 4 have to be approached very carefully. For all studies analyzed here, it is possible that appetite assessments might not have been administered early enough in the course of treatment to capture meaningful changes; our earliest measurements are at 2 weeks, but changes in appetite might have occurred as early as Day 1 of treatment, and by 2 weeks weight changes were as informative as appetite changes. Additionally, the use of different appetite assessment scales limits comparisons across studies and most of the appetite scales used here have not been validated. Within each study, appetite assessment scales were administered repeatedly to all patients, which might have desensitized the scales and resulted in a loss of accuracy. Also, the analyses were not adjusted for baseline psychopathology in the different patient groups and for dose of olanzapine. Finally, the cutoffs to define patient groups that experienced appetite scale score increases, no change, or decreases were based on clinical experience, but without access to previous reports in the literature to guide this decision. Future research is warranted to further assess the validity of the chosen cutoffs.

## Conclusion

In conclusion, no consistent correlation between changes in appetite and weight changes could be observed in our analysis. However, when it was present, it was in the expected direction, and the trend was consistently in the expected direction. Consequently, appetite change should be considered in patient care, but when regular weight monitoring is performed, appetite does not add additional information predicting future weight changes during treatment with olanzapine: early weight change may be a more useful predictor for long-term weight change. Patients who experience early weight gain or are otherwise at risk for significant weight gain during olanzapine treatment should receive regular monitoring of weight and lifestyle educational programs early in the course of illness and of treatment.

## Competing interests

This work was sponsored by Eli Lilly and Company and/or any of its subsidiaries. Drs. Karagianis, Treuer, and Hoffmann and Mr. Case are full-time employees and minor stockholders of Eli Lilly and Company and/or any of its subsidiaries.

## Authors' contributions

MC was involved in the design of the study, performed the statistical analyses, and revised the manuscript. TT and JK conducted the clinical studies, were involved in study design, contributed to interpreting the results in a clinical context, and revised the manuscript. VPH was involved in study design and conduct of the clinical studies, in design of the current analyses, contributed to interpreting the results in a clinical context, and revised the manuscript. All authors read and approved the final manuscript.

## Pre-publication history

The pre-publication history for this paper can be accessed here:

http://www.biomedcentral.com/1471-244X/10/72/prepub
